# Supplementary material for: Evidence for deleterious effects of immunological history in SARS-CoV-2
Source: PLoS One. 2022 Aug 24;17(8):e0272163. doi: 10.1371/journal.pone.0272163 (PMC9401162; doi:10.1371/journal.pone.0272163)
Supplement: S1 File — (PDF) [file pone.0272163.s001.pdf]

SWISS-MODEL Homology Modelling Report

Model Building Report

This document lists the results for the homology modelling project "Untitled Project" submitted to SWISS-MODEL workspace on March 30, 2021, 10:35 a.m..The submitted primary amino acid sequence is given in Table T1.

If you use any results in your research, please cite the relevant publications:

- Waterhouse, A., Bertoni, M., Bienert, S., Studer, G., Tauriello, G., Gumienny, R., Heer, F.T., de Beer, T.A.P., Rempfer, C., Bordoli, L., Lepore, R., Schwede, T. SWISS-MODEL: homology modelling of protein structures and complexes. Nucleic Acids Res. 46(W1), W296-W303 (2018). doi>
- Bienert, S., Waterhouse, A., de Beer, T.A.P., Tauriello, G., Studer, G., Bordoli, L., Schwede, T. The SWISS-MODEL Repository - new features and functionality. Nucleic Acids Res. 45, D313-D319 (2017). doi>
- Studer, G., Tauriello, G., Bienert, S., Biasini, M., Johner, N., Schwede, T. ProMod3 - A versatile homology modelling toolbox. PLOS Comp. Biol. 17(1), e1008667 (2021). doi>
- Studer, G., Rempfer, C., Waterhouse, A.M., Gumienny, G., Haas, J., Schwede, T. QMEANDisCo - distance constraints applied on model quality estimation. Bioinformatics 36, 1765-1771 (2020). doi>
- Bertoni, M., Kiefer, F., Biasini, M., Bordoli, L., Schwede, T. Modeling protein quaternary structure of homo- and hetero-oligomers beyond binary interactions by homology. Scientific Reports 7 (2017). doi>

Results

The SWISS-MODEL template library (SMTL version 2021-03-25, PDB release 2021-03-19) was searched with BLAST (Camacho et al.) and HHblits (Steinegger et al.) for evolutionary related structures matching the target sequence in Table T1. For details on the template search, see Materials and Methods. Overall 189 templates were found (Table T2).

Models

The following model was built (see Materials and Methods "Model Building"):

Model #01	File	Built with	Oligo-State	Ligands	GMQE	QMEAN
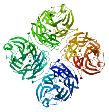	PDB	ProMod3 3.2.0	homo-tetramer (matching prediction)	4 x CA: CALCIUM ION;<br>1 x NAG: 2-acetamido-2-deoxy-beta-D-glucopyranose;<br>1 x NAG-FUC: alpha-L-fucopyranose-(1-6)-2-acetamido-2-deoxy-beta-D-glucopyranose;	0.95	-1.40

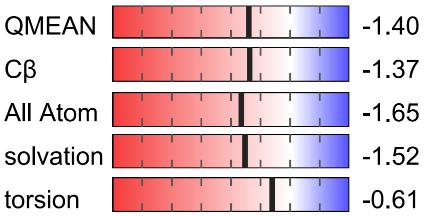

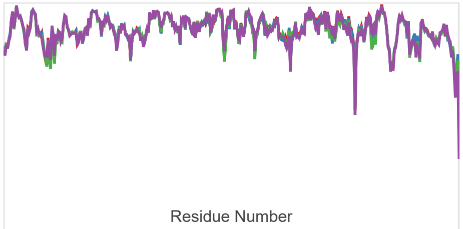

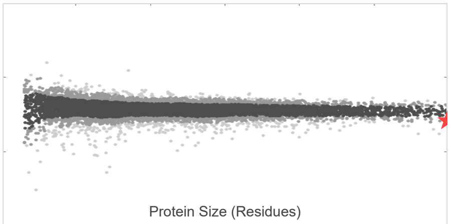

Template	Seq Identity	Oligo-state	QSQE	Found by	Method	Resolution	Seq Similarity	Range	Coverage	Description
4gzs.1.A	96.39	homo-tetramer	1.00	HHblits	X-ray	2.35Å	0.62	1 - 360	1.00	Neuraminidase

Included Ligands

Ligand	Description
4 x CA	CALCIUM ION
1 x NAG	2-acetamido-2-deoxy-beta-D-glucopyranose
1 x NAG-FUC	alpha-L-fucopyranose-(1-6)-2-acetamido-2-deoxy-beta-D-glucopyranose

Excluded ligands

Ligand Name.Number	Reason for Exclusion	Description
CA.16	Not in contact with model.	CALCIUM ION
EPE.10	Not biologically relevant.	4-(2-HYDROXYETHYL)-1-PIPERAZINE ETHANESULFONIC ACID
EPE.13	Not biologically relevant.	4-(2-HYDROXYETHYL)-1-PIPERAZINE ETHANESULFONIC ACID
EPE.18	Not biologically relevant.	4-(2-HYDROXYETHYL)-1-PIPERAZINE ETHANESULFONIC ACID
EPE.22	Not biologically relevant.	4-(2-HYDROXYETHYL)-1-PIPERAZINE ETHANESULFONIC ACID
NAG.11	Clashing with protein.	2-acetamido-2-deoxy-beta-D-glucopyranose
NAG.14	Clashing with protein.	2-acetamido-2-deoxy-beta-D-glucopyranose
NAG.15	Clashing with protein.	2-acetamido-2-deoxy-beta-D-glucopyranose
NAG.19	Clashing with protein.	2-acetamido-2-deoxy-beta-D-glucopyranose
NAG.20	Not in contact with model.	2-acetamido-2-deoxy-beta-D-glucopyranose
NAG.24	Clashing with protein.	2-acetamido-2-deoxy-beta-D-glucopyranose
NAG-FUC.2	Clashing with protein.	alpha-L-fucopyranose-(1-6)-2-acetamido-2-deoxy-beta-D-glucopyranose
NAG-FUC.4	Clashing with protein.	alpha-L-fucopyranose-(1-6)-2-acetamido-2-deoxy-beta-D-glucopyranose
NAG-NAG.8	Not in contact with model.	2-acetamido-2-deoxy-beta-D-glucopyranose-(1-4)-2-acetamido-2-deoxy-beta-D-glucopyranose
NAG-NAG-BMA-MAN-MAN.1	Clashing with protein.	alpha-D-mannopyranose-(1-3)-[alpha-D-mannopyranose-(1-6)]beta-D-mannopyranose-(1-4)-2-acetamido-2-deoxy-beta-D-glucopyranose-(1-4)-2-acetamido-2-deoxy-beta-D-glucopyranose
NAG-NAG-BMA-MAN-MAN.3	Clashing with protein.	alpha-D-mannopyranose-(1-3)-[alpha-D-mannopyranose-(1-6)]beta-D-mannopyranose-(1-4)-2-acetamido-2-deoxy-beta-D-glucopyranose-(1-4)-2-acetamido-2-deoxy-beta-D-glucopyranose
NAG-NAG-BMA-MAN-MAN.5	Clashing with protein.	alpha-D-mannopyranose-(1-3)-[alpha-D-mannopyranose-(1-6)]beta-D-mannopyranose-(1-4)-2-acetamido-2-deoxy-beta-D-glucopyranose-(1-4)-2-acetamido-2-deoxy-beta-D-glucopyranose
NAG-NAG-BMA-MAN-MAN.7	Not biologically relevant.	alpha-D-mannopyranose-(1-3)-[alpha-D-mannopyranose-(1-6)]beta-D-mannopyranose-(1-4)-2-acetamido-2-deoxy-beta-D-glucopyranose-(1-4)-2-acetamido-2-deoxy-beta-D-glucopyranose

```
Target    AGGDIWVTREPYVSCDPDKGNQFALGQGTTLSKGHSNNTVRDRTPYRTLLMNELGVPFHLGTKQVCIAWSSSSCHDGKAW
4gzs.1.A  AGGDIWVTREPYVSCDPDKCYQFALGQGTTLNNVHSNNTVRGRTPYRTLLMNELGVPFHLGTKQVCIAWSSSSCHDGKAW

Target    LHVCITGDDKNATASFIYNGRLVDSVVSWSKEVLRTQESECVCINGTCTVVMTDGSASGKADTKILFIEEGKIVHTSTLS
4gzs.1.A  LHVCITGDDKNATASFIYNGRLVDSVVSWSKEILRTQESECVCINGTCTVVMTDGSASGKADTKILFIEEGKIVHTSTLS

Target    GSAQHVEECSCYPRYPGVRCVCRDNWKGSNRPIVDINIKDHSIVSSYVCSGLVGDTPRKNDSSSSGHCLNPNNEEGGHGV
4gzs.1.A  GSAQHVEECSCYPRYPGVRCVCRDNWKGSNRPIVDINIKDHSIVSSYVCSGLVGDTPRKNDSSSSSHCLDPNNEEGGHGV

Target    KGWAFDDGNDVWMGRTINETSRLGYETFKVIEGWSNPKSKLQINRQVIVDRGDRSGYSGIFSVEGKSCINRCFYVELIRG
4gzs.1.A  KGWAFDDGNDVWMGRTINETSRLGYETFKVIEGWSNPKSKLQINRQVIVDRGNRSGYSGIFSVEGKSCINRCFYVELIRG

Target    RKEETEVLWTSNSILLFCGTSGTYGTGSWPDAADLNLMPI
4gzs.1.A  RKEETEVLWTSNSIVVFCGTSGTYGTGSWPDGADLNLMPI
```

```
Target    AGGDIWVTREPYVSCDPDKGNQFALGQGTTLSKGHSNNTVRDRTPYRTLLMNELGVPFHLGTKQVCIAWSSSSCHDGKAW
4gzs.1.B  AGGDIWVTREPYVSCDPDKCYQFALGQGTTLNNVHSNNTVRGRTPYRTLLMNELGVPFHLGTKQVCIAWSSSSCHDGKAW

Target    LHVCITGDDKNATASFIYNGRLVDSVVSWSKEVLRTQESECVCINGTCTVVMTDGSASGKADTKILFIEEGKIVHTSTLS
4gzs.1.B  LHVCITGDDKNATASFIYNGRLVDSVVSWSKEILRTQESECVCINGTCTVVMTDGSASGKADTKILFIEEGKIVHTSTLS

Target    GSAQHVEECSCYPRYPGVRCVCRDNWKGSNRPIVDINIKDHSIVSSYVCSGLVGDTPRKNDSSSSGHCLNPNNEEGGHGV
4gzs.1.B  GSAQHVEECSCYPRYPGVRCVCRDNWKGSNRPIVDINIKDHSIVSSYVCSGLVGDTPRKNDSSSSSHCLDPNNEEGGHGV

Target    KGWAFDDGNDVWMGRTINETSRLGYETFKVIEGWSNPKSKLQINRQVIVDRGDRSGYSGIFSVEGKSCINRCFYVELIRG
4gzs.1.B  KGWAFDDGNDVWMGRTINETSRLGYETFKVIEGWSNPKSKLQINRQVIVDRGNRSGYSGIFSVEGKSCINRCFYVELIRG

Target    RKEETEVLWTSNSILLFCGTSGTYGTGSWPDAADLNLMPI
4gzs.1.B  RKEETEVLWTSNSIVVFCGTSGTYGTGSWPDGADLNLMPI
```

```
Target    AGGDIWVTREPYVSCDPDKGNQFALGQGTTLSKGHSNNTVRDRTPYRTLLMNELGVPFHLGTKQVCIAWSSSSCHDGKAW
4gzs.1.C  AGGDIWVTREPYVSCDPDKCYQFALGQGTTLNNVHSNNTVRGRTPYRTLLMNELGVPFHLGTKQVCIAWSSSSCHDGKAW

Target    LHVCITGDDKNATASFIYNGRLVDSVVSWSKEVLRTQESECVCINGTCTVVMTDGSASGKADTKILFIEEGKIVHTSTLS
4gzs.1.C  LHVCITGDDKNATASFIYNGRLVDSVVSWSKEILRTQESECVCINGTCTVVMTDGSASGKADTKILFIEEGKIVHTSTLS

Target    GSAQHVEECSCYPRYPGVRCVCRDNWKGSNRPIVDINIKDHSIVSSYVCSGLVGDTPRKNDSSSSGHCLNPNNEEGGHGV
4gzs.1.C  GSAQHVEECSCYPRYPGVRCVCRDNWKGSNRPIVDINIKDHSIVSSYVCSGLVGDTPRKNDSSSSSHCLDPNNEEGGHGV

Target    KGWAFDDGNDVWMGRTINETSRLGYETFKVIEGWSNPKSKLQINRQVIVDRGDRSGYSGIFSVEGKSCINRCFYVELIRG
4gzs.1.C  KGWAFDDGNDVWMGRTINETSRLGYETFKVIEGWSNPKSKLQINRQVIVDRGNRSGYSGIFSVEGKSCINRCFYVELIRG

Target    RKEETEVLWTSNSILLFCGTSGTYGTGSWPDAADLNLMPI
4gzs.1.C  RKEETEVLWTSNSIVVFCGTSGTYGTGSWPDGADLNLMPI
```

```
Target    AGGDIWVTREPYVSCDPDKGNQFALGQGTTLSKGHSNNTVRDRTPYRTLLMNELGVPFHLGTKQVCIAWSSSSCHDGKAW
4gzs.1.D  AGGDIWVTREPYVSCDPDKCYQFALGQGTTLNNVHSNNTVRGRTPYRTLLMNELGVPFHLGTKQVCIAWSSSSCHDGKAW

Target    LHVCITGDDKNATASFIYNGRLVDSVVSWSKEVLRTQESECVCINGTCTVVMTDGSASGKADTKILFIEEGKIVHTSTLS
4gzs.1.D  LHVCITGDDKNATASFIYNGRLVDSVVSWSKEILRTQESECVCINGTCTVVMTDGSASGKADTKILFIEEGKIVHTSTLS

Target    GSAQHVEECSCYPRYPGVRCVCRDNWKGSNRPIVDINIKDHSIVSSYVCSGLVGDTPRKNDSSSSGHCLNPNNEEGGHGV
4gzs.1.D  GSAQHVEECSCYPRYPGVRCVCRDNWKGSNRPIVDINIKDHSIVSSYVCSGLVGDTPRKNDSSSSSHCLDPNNEEGGHGV

Target    KGWAFDDGNDVWMGRTINETSRLGYETFKVIEGWSNPKSKLQINRQVIVDRGDRSGYSGIFSVEGKSCINRCFYVELIRG
4gzs.1.D  KGWAFDDGNDVWMGRTINETSRLGYETFKVIEGWSNPKSKLQINRQVIVDRGNRSGYSGIFSVEGKSCINRCFYVELIRG

Target    RKEETEVLWTSNSILLFCGTSGTYGTGSWPDAADLNLMPI
4gzs.1.D  RKEETEVLWTSNSIVVFCGTSGTYGTGSWPDGADLNLMPI
```

Materials and Methods

Template Search

Template search with BLAST and HHblits has been performed against the SWISS-MODEL template library (SMTL, last update: 2021-03-25, last included PDB release: 2021-03-19).

The target sequence was searched with BLAST against the primary amino acid sequence contained in the SMTL. A total of 103 templates were found.

An initial HHblits profile has been built using the procedure outlined in (Steinegger et al.), followed by 1 iteration of HHblits against Uniclust30 (Mirdita, von den Driesch et al.). The obtained profile has then be searched against all profiles of the SMTL. A total of 104 templates were found.

Template Selection

For each identified template, the template's quality has been predicted from features of the target-template alignment. The templates with the highest quality have then been selected for model building.

Model Building

Models are built based on the target-template alignment using ProMod3 (Studer et al.). Coordinates which are conserved between the target and the template are copied from the template to the model. Insertions and deletions are remodelled using a fragment library. Side chains are then rebuilt. Finally, the geometry of the resulting model is regularized by using a force field.

Model Quality Estimation

The global and per-residue model quality has been assessed using the QMEAN scoring function (Studer et al.).

Ligand Modelling

Ligands present in the template structure are transferred by homology to the model when the following criteria are met: (a) The ligands are annotated as biologically relevant in the template library, (b) the ligand is in contact with the model, (c) the ligand is not clashing with the protein, (d) the residues in contact with the ligand are conserved between the target and the template. If any of these four criteria is not satisfied, a certain ligand will not be included in the model. The model summary includes information on why and which ligand has not been included.

Oligomeric State Conservation

The quaternary structure annotation of the template is used to model the target sequence in its oligomeric form. The method (Bertoni et al.) is based on a supervised machine learning algorithm, Support Vector Machines (SVM), which combines interface conservation, structural clustering, and other template features to provide a quaternary structure quality estimate (QSQE). The QSQE score is a number between 0 and 1, reflecting the expected accuracy of the interchain contacts for a model built based a given alignment and template. Higher numbers indicate higher reliability. This complements the GMQE score which estimates the accuracy of the tertiary structure of the resulting model.

References

- **BLAST**
  Camacho, C., Coulouris, G., Avagyan, V., Ma, N., Papadopoulos, J., Bealer, K., Madden, T.L. BLAST+: architecture and applications. BMC Bioinformatics 10, 421-430 (2009).
- **HHblits**
  Steinegger, M., Meier, M., Mirdita, M., Vöhringer, H., Haunsberger, S. J., Söding, J. HH-suite3 for fast remote homology detection and deep protein annotation. BMC Bioinformatics 20, 473 (2019).
- **Uniclust30**
  Mirdita, M., von den Driesch, L., Galiez, C., Martin, M.J., Söding, J., Steinegger, M. Uniclust databases of clustered and deeply annotated protein sequences and alignments. Nucleic Acids Research 45, D170–D176 (2016).

Table T1:

Primary amino acid sequence for which templates were searched and models were built.

```
AGGDIWVTREPYVSCDPDKGNQFALGQGTTLSKGHSNNTVRDRTPYRTLLMNELGVPFHLGTKQVCIAWSSSSCHDGKAWLHVCITGDDKNATASFIYNG
RLVDSVVSWSKEVLRTQESECVCINGTCTVVMTDGSASGKADTKILFIEEGKIVHTSTLSGSAQHVEECSCYPRYPGVRCVCRDNWKGSNRPIVDINIKD
HSIVSSYVCSGLVGDTPRKNDSSSSGHCLNPNNEEGGHGVKGWAFDDGNDVWMGRTINETSRLGYETFKVIEGWSNPKSKLQINRQVIVDRGDRSGYSGI
FSVEGKSCINRCFYVELIRGRKEETEVLWTSNSILLFCGTSGTYGTGSWPDAADLNLMPI
```

Table T2:

Template	Seq Identity	Oligo-state	QSQE	Found by	Method	Resolution	Seq Similarity	Coverage	Description
4gzs.1.A	96.39	homo-tetramer	1.00	HHblits	X-ray	2.35Å	0.62	1.00	Neuraminidase
4gzo.1.A	96.67	homo-tetramer	1.00	HHblits	X-ray	2.60Å	0.62	1.00	neuraminidase

Template	Seq Identity	Oligo-state	QSQE	Found by	Method	Resolution	Seq Similarity	Coverage	Description
3tia.1.A	82.78	homo-tetramer	1.00	HHblits	X-ray	1.80Å	0.57	1.00	Neuraminidase
4k1j.1.A	82.78	homo-tetramer	1.00	HHblits	X-ray	2.20Å	0.57	1.00	Neuraminidase
4h52.1.A	82.78	homo-tetramer	1.00	HHblits	X-ray	1.80Å	0.57	1.00	Neuraminidase
5huk.1.A	79.72	homo-tetramer	1.00	HHblits	X-ray	2.45Å	0.57	1.00	Neuraminidase
6n4d.1.A	79.17	homo-tetramer	1.00	HHblits	X-ray	1.80Å	0.56	1.00	Neuraminidase
2bat.1.A	83.06	homo-tetramer	1.00	HHblits	X-ray	2.00Å	0.57	1.00	NEURAMINIDASE N2
1ivg.1.A	82.78	homo-tetramer	1.00	HHblits	X-ray	1.90Å	0.57	1.00	INFLUENZA A SUBTYPE N2 NEURAMINIDASE
1ive.1.A	82.78	homo-tetramer	1.00	HHblits	X-ray	2.40Å	0.57	1.00	INFLUENZA A SUBTYPE N2 NEURAMINIDASE
1inh.1.A	82.78	homo-tetramer	1.00	HHblits	X-ray	2.40Å	0.57	1.00	INFLUENZA A SUBTYPE N2 NEURAMINIDASE
1ing.1.A	82.78	homo-tetramer	1.00	HHblits	X-ray	2.40Å	0.57	1.00	INFLUENZA A SUBTYPE N2 NEURAMINIDASE
6br5.1.A	95.83	monomer	-	HHblits	X-ray	2.04Å	0.62	1.00	Neuraminidase
2aep.1.A	90.56	homo-tetramer	1.00	HHblits	X-ray	2.10Å	0.60	1.00	neuraminidase
6n6b.1.A	89.17	homo-tetramer	1.00	HHblits	X-ray	2.30Å	0.60	1.00	Neuraminidase
4mwx.1.B	47.62	homo-tetramer	0.86	HHblits	X-ray	1.80Å	0.44	0.99	Neuraminidase
4mwj.1.A	47.90	homo-tetramer	0.85	HHblits	X-ray	1.80Å	0.44	0.99	Neuraminidase
5nz4.1.A	46.05	homo-tetramer	0.88	HHblits	X-ray	1.36Å	0.43	0.98	neuraminidase
2b8h.1.A	47.62	homo-tetramer	0.85	HHblits	X-ray	2.20Å	0.44	0.99	Neuraminidase
4m3m.1.A	44.94	homo-tetramer	0.87	HHblits	X-ray	2.10Å	0.43	0.99	Neuraminidase
5l14.1.A	47.90	homo-tetramer	0.84	HHblits	X-ray	1.90Å	0.44	0.99	Neuraminidase
4mwj.1.A	49.72	homo-tetramer	0.84	BLAST	X-ray	1.80Å	0.45	0.98	Neuraminidase
2b8h.1.A	49.43	homo-tetramer	0.84	BLAST	X-ray	2.20Å	0.45	0.98	Neuraminidase
1nna.1.D	49.15	homo-tetramer	0.84	BLAST	X-ray	2.50Å	0.44	0.98	NEURAMINIDASE
4mju.1.A	45.51	homo-tetramer	0.84	HHblits	X-ray	2.35Å	0.43	0.99	Neuraminidase
4nn9.1.A	49.15	homo-tetramer	0.83	BLAST	X-ray	2.30Å	0.44	0.98	NEURAMINIDASE N9
1mwe.1.C	49.15	homo-tetramer	0.83	BLAST	X-ray	1.70Å	0.44	0.98	NEURAMINIDASE
6pzw.1.A	47.90	homo-tetramer	0.87	HHblits	EM	NA	0.44	0.99	Neuraminidase
6pze.1.A	47.90	homo-tetramer	0.83	HHblits	X-ray	2.30Å	0.44	0.99	Neuraminidase
6pze.1.A	49.72	homo-tetramer	0.83	BLAST	X-ray	2.30Å	0.45	0.98	Neuraminidase

Template	Seq Identity	Oligo-state	QSQE	Found by	Method	Resolution	Seq Similarity	Coverage	Description
6pzf.1.A	47.90	homo-tetramer	0.84	HHblits	X-ray	2.80Å	0.44	0.99	Neuraminidase
1nca.2.C	49.15	homo-tetramer	0.84	BLAST	X-ray	2.50Å	0.44	0.98	INFLUENZA A SUBTYPE N9 NEURAMINIDASE
1ncd.1.A	47.90	homo-tetramer	0.84	HHblits	X-ray	2.90Å	0.44	0.99	INFLUENZA A SUBTYPE N9 NEURAMINIDASE
1nmb.1.A	47.90	homo-tetramer	0.81	HHblits	X-ray	2.20Å	0.44	0.99	N9 NEURAMINIDASE
4d8s.1.A	45.79	homo-tetramer	0.88	HHblits	X-ray	2.40Å	0.43	0.99	Neuraminidase
4gb1.1.A	45.79	homo-tetramer	0.85	HHblits	X-ray	2.62Å	0.43	0.99	Neuraminidase
4b7q.1.A	46.18	homo-tetramer	0.85	HHblits	X-ray	2.73Å	0.43	0.98	NEURAMINIDASE
3o9j.1.A	45.92	homo-tetramer	0.84	HHblits	X-ray	2.00Å	0.43	0.99	Neuraminidase
4mjv.1.A	45.79	homo-tetramer	0.83	HHblits	X-ray	2.65Å	0.43	0.99	Neuraminidase
2ht7.1.A	45.51	homo-tetramer	0.81	HHblits	X-ray	2.60Å	0.43	0.99	Neuraminidase
4ks5.1.A	44.94	homo-tetramer	0.77	HHblits	X-ray	2.70Å	0.43	0.99	Neuraminidase
6crd.1.D	49.15	homo-tetramer	0.75	BLAST	X-ray	2.57Å	0.44	0.98	Tetrabrachion,Neuraminidase
6crd.1.B	49.15	homo-tetramer	0.75	BLAST	X-ray	2.57Å	0.44	0.98	Tetrabrachion,Neuraminidase
6crd.1.C	49.15	homo-tetramer	0.75	BLAST	X-ray	2.57Å	0.44	0.98	Tetrabrachion,Neuraminidase
1ncc.1.A	49.15	homo-tetramer	0.86	BLAST	X-ray	2.50Å	0.44	0.98	INFLUENZA A SUBTYPE N9 NEURAMINIDASE
1ncd.1.A	49.43	homo-tetramer	0.83	BLAST	X-ray	2.90Å	0.45	0.98	INFLUENZA A SUBTYPE N9 NEURAMINIDASE
1nmb.1.A	49.43	homo-tetramer	0.81	BLAST	X-ray	2.20Å	0.45	0.98	N9 NEURAMINIDASE
6lxk.1.A	48.13	homo-tetramer	0.84	BLAST	X-ray	3.61Å	0.44	0.96	Neuraminidase
1nma.1.A	47.62	homo-tetramer	0.68	HHblits	X-ray	3.00Å	0.44	0.99	N9 NEURAMINIDASE
1nma.1.A	49.43	homo-tetramer	0.65	BLAST	X-ray	3.00Å	0.45	0.98	N9 NEURAMINIDASE

The table above shows the top 50 filtered templates. A further 83 templates were found which were considered to be less suitable for modelling than the filtered list.
1a4g.1.A, 1b9v.1.D, 1inf.1.A, 1iny.1.A, 1l7g.1.A, 1mwe.1.C, 1nca.2.C, 1ncb.1.A, 1ncc.1.A, 1nna.1.D, 1vcj.1.A, 2ht7.1.A, 2htv.1.A, 2hu0.1.A, 2qwd.1.A, 3b7e.1.A, 3cl0.1.A, 3cl2.1.A, 3cye.1.A, 3f14.1.A, 3k36.1.A, 3k38.1.A, 3nn9.1.A, 3o9j.1.A, 3sal.1.A, 4b7m.1.C, 4b7q.1.A, 4b7r.1.A, 4cpl.1.A, 4cpo.1.A, 4d8s.1.A, 4fvk.1.A, 4gb1.1.A, 4gdi.1.A, 4gdi.1.B, 4gdj.1.A, 4gez.1.A, 4h53.1.A, 4h53.1.D, 4hzv.1.A, 4hzy.1.A, 4hzz.1.A, 4k3y.1.A, 4k3y.1.C, 4ks5.1.A, 4m3m.1.A, 4mc7.1.A, 4mju.1.A, 4mjv.1.A, 4mwx.1.B, 4nn9.1.A, 4qn3.1.A, 4qn4.1.A, 4qnp.1.A, 4wa5.1.D, 5hug.1.A, 5hum.1.A, 5hun.1.A, 5l14.1.A, 5nn9.1.A, 5nwe.1.A, 5nz4.1.A, 5nze.1.A, 5nzf.1.A, 5nzn.1.A, 6crd.1.A, 6crd.1.B, 6crd.1.C, 6crd.1.D, 6d96.1.A, 6g01.2.D, 6hfy.1.D, 6hp0.1.C, 6lxi.1.A, 6lxk.1.A, 6nn9.1.A, 6pzf.1.A, 6pzw.1.A, 6q20.1.A, 6q23.1.A, 6v4n.1.A, 6v4o.1.A, 7cm1.1.A
