# Supplementary material for: Evidence for deleterious effects of immunological history in SARS-CoV-2
Source: PLoS One. 2022 Aug 24;17(8):e0272163. doi: 10.1371/journal.pone.0272163 (PMC9401162; doi:10.1371/journal.pone.0272163)
Supplement: S1 Table — (PDF) [file pone.0272163.s002.pdf]

## S1 Table: Potential primary epitopes targeted by αEp9 Abs

| Construct # | Pathogen | Target protein | Accession No. | Residues | Epitope sequences | Similarity % |
|---|---|---|---|---|---|---|
| **Phage-displayed constructs** | | | | | | **template: Ep9 or EpNeu** |
| 1 | SARS-COV-2 (Ep9) | Nucleocapsid | QQX29443.1 | 152-172 | ANNAAIVLQLPQGTTLPKGFY | - |
| 2 | SARS-COV-1 | Nucleocapsid | YP_009825061.1 | 153-173 | NNNAATVLQLPQGTTLPKGFY | **90.5** |
| 3 | MERS | Nucleocapsid | YP_009047211.1 | 141-151 | NNDSAIVTQFAPGTKLPKNFH | **66.7** |
| 4 | Human coronavirus HKU1 | Nucleocapsid | YP_173242.1 | 166-186 | TTQEAIPTRFPPGTILPQGYY | **57.1** |
| 5 | Human coronavirus NL63 | Nucleocapsid | YP_003771.1 | 119-136 | NQKPLEPKFSIALPPELS | **13.8** |
| 6 | Human coronavirus OC43 | Nucleocapsid | YP_009555245.1 | 167-187 | SSDEAIPTRFPPGTVLPQGYY | **71.4** |
| 7 | Human coronavirus 229E | Nucleocapsid | AGW80953.1 | 122-138 | SEPEIPHFNQKLPNGVT | **21.4** |
| 8 | Human adenovirus 61 | Hexon | AQQ81927.1 | 123-164 | ANNAATPQVVFYTEDVNLEMPDTHLVFKPAVPNGTIASESLL | **17.6** |
| 9 | Human mastadenovirus E | PVIII | YP_068038.1 | 76-114 | AALVYQEIPQPTTVLLPRDAQAEVQLTNSGVQLAGGATL | **31** |
| 10 | Influenza A virus (A/Utah/40/2017) | PB2 polymerase | AVH77902.1 | 225-244 | GSVYIEVLHLTQGTCWEQMY | **41.7** |
| 11 | Influenza A virus (**EpNeu**) (A/Para/128982-IEC/2014(H3N2) | Neuraminidase, partial | AIX95025.1 | 34-46 | ALGQGTTLSKGHS | **38.1** |
| 12 | Influenza B virus (B/California/88/2019) | Neuraminidase | QIA55965.1 | 67-79 | ATKGVVLLLPEPE | **28.6** |
| 13 | Influenza C virus (C/Singapore/DSO-070193/2006) | Polymerase PB1, partial | AFV68302.1 | 119-145 | AATALQLTVDAIKETEGPFKGTTMLEY | **34.4** |
| 14 | Human respiratory syncytial virus A | Fusion protein | ASU44644.1 | 87-100 | NNAVTELQLLMQST | **38.1** |
| 15 | Human respiratory syncytial virus A | Attachment glycoprotein | ART28426.1 | 106-116 | GTTPQSTTIPA | **28.6** |
| 16 | Human metapneumovirus | Nucleoprotein, partial | ABO15448.1 | 11-33 | TTTAVTPSSLPQEITLLCGEILY | **34.8** |
| 17 | Human metapneumovirus | Attachment glycoprotein, partial | AEW90340.1 | 57-72 | PQQTTDKHTALPKSIY | **30.8** |
| 18 | Human betaherpesvirus 6A | Immediate Early protein 2 | AGJ52064.1 | 1396-1422 | AATPIDFVGAVKTCNKYAKDNPKEIVL | **10** |
| 19 | Verrucomicrobia bacterium | NADH-quinone oxidoreductase (NOX) | PYJ45937.1 | 76-89 | AGVVLQLPQGTTL | **57.1** |

| | | | | | | |
|---|---|---|---|---|---|---|
| 20 | Clostridium butyricum | Bifunctional methylenetetrahydrofolate dehydrogenase/methenyltetrahydrofolate cyclohydrolase (MTHFD2) | MBE6063617.1 | 94-104 | IILQLPLPKKF | **47.6** |
| 21 | Fusobacterium mortiferum | Type II secretion protein | WP_118233983.1 | 77-99 | VENGAIVLQYDKEIYLGLTENFF | **48** |
| 22 | Fusobacterium mortiferum | Autotransporter outer membrane protein | WP_005886362.1 | 449-460 | NGAIVGDLVQGT | **38.1** |
| 23 | Influenza A virus (A/swine/Missouri/A01774733/2016(H1N2) or A/Para/129501-IEC/2014(H3N2)) | Neuraminidase | ANK78229.1 / AIX95013 | 133-145/ 24-36 | ALGQGTTLSNGHS | **92.3** |
| 24 | Influenza A (A/swine/Minnesota/A01394278/2013(H3N2)) | Neuraminidase | AHA57095.1 | 134-146 | ALGQGTTLNNGHS | **92.3** |
| 25 | Influenza A virus (A/California/04/2009 (H1N1)) | Neuraminidase | AJI76397.1 | 75-89 | TFFLTQGALLNDKHS | **46.7** |
| 26 | Influenza A virus (A/California/111/2015(H3N2)) | Neuraminidase | ANM97445.1 | 133-145 | ALGQGTTLNNVHS | **84.6** |
| 27 | Influenza A virus (A/mallard/California/1156/2010(H4N6)) | Neuraminidase | AEK50939.1 | 133-142 | ALSQGTTLKG | **84.6** |
| 28 | Influenza A virus (A/California/33/2011(H3N2)) | Neuramidase | AGL06761.1 | 133-145 | ALGQGTTLSNVHS | **84.6** |
| 29 | Influenza A virus (**EpNeu Pred**) (A/Para/128982-IEC/2014(H3N2)) | Neuraminidase, partial | AIX95025.1 | 34-56 | ALGQGTTLSKGHSNNTVRDRTPY | **-** |
| **eGFP-fusion constructs** | | | | | | |
| 30 | Influenza A virus (**EpNeu**) (A/Para/128982-IEC/2014(H3N2)) | Neuraminidase | AIX95025.1 | 34-46 | ALGQGTTLSKGHS | **-** |
| 31 | Influenza A virus (**EpNeu Pred**) (A/Para/128982-IEC/2014(H3N2)) | Neuraminidase | AIX95025.1 | 34-56 | ALGQGTTLSKGHSNNTVRDRTPY | **-** |
