# Supplementary material for: Evidence for deleterious effects of immunological history in SARS-CoV-2
Source: PLoS One. 2022 Aug 24;17(8):e0272163. doi: 10.1371/journal.pone.0272163 (PMC9401162; doi:10.1371/journal.pone.0272163)
Supplement: S2 Table — (PDF) [file pone.0272163.s003.pdf]

S2 Table: Primers used to subclone potential original epitopes

Construct #	Primer names:	Oligonucleotide sequence
**Phage-displayed constructs**		
1	primer_1_F	tcaagggactaccttgcccaaggggttctatGGTGGAGGATCCGGGAGC
	primer_1_R	ggtaattgtaacacgattgcagcgttattagcTCCACTTCCTTTATCATCGTCATCTTTATAATC
2	primer_2_F	acttccacagggaacgacactgccaaagggatttGGTGGAGGATCCGGGAGC
	primer_2_R	tgcagtacagtggcagcattgttatttccacttccTTTATCATCGTCATCTTTATAATCAACCAATGC
3	primer_3_F	tccgggtacaaagttaccaaagaacttccacGGTGGAGGATCCGGGAGC
	primer_3_R	gcaaattgagtaactatcgctgaatcattgttTCCACTTCCTTTATCATCGTCATCTTTATAATC
4	primer_4_F	tcccggaactattttaccccaaggatactatGGTGGAGGATCCGGGAGC
	primer_4_R	gggaatctagtgggaatcgcctcctgagtagtTCCACTTCCTTTATCATCGTCATCTTTATAATC
5	primer_5_F	agtattgccttgccacctgagttatctGGTGGAGGATCCGGGAGC
	primer_5_R	aaatttcggttcaagcggcttttgattTCCACTTCCTTTATCATCGTCATCTTTATAATC
6	primer_6_F	tccgggtactgttttgcctcagggttactacGGTGGAGGATCCGGGAGC
	primer_6_R	ggaaaccgcgttgggatggcctcatcagagctTTTATCATCGTCATCTTTATAATCAACCAATGC
7	primer_7_F	ccagaaattgccgaacggcgttactGGTGGAGGATCCGGGAGC
	primer_7_R	ttaaagtgagggatctctggctcgctTTTATCATCGTCATCTTTATAATCAACCAATGC
8	primer_8_F	TTCTAAATTCACATCCTCAGTATAGAACACGACTTGTGGGGTTGCTGCATTGTTGGCTTTATCATCGTCATCTTTATAATCAACCAATGC
	primer_8_R	ATGCCAGATACTCATTTGGTCTTCAAACCTGCGGTCCCGAATGGCACGATTGCTAGTGAATCTCTCCTCggtggaggatccgggagc
9	primer_9_F	ACGCGGGAGCAGCACTGTAGTCGGCTGAGGGATTTCCTGGTACACCAGGGCGGCTTTATCATCGTCATCTTTATAATCAACCAATGC
	primer_9_R	GACGCGCAAGCCGAGGTCCAGTTGACAAACAGCGGTGTACAATTGGCTGGCGGTGCCACTCTGggtggaggatccgggagc
10	primer_10_F	acacaaggcacttgctgggagcaaatgtatGGTGGAGGATCCGGGAGC
	primer_10_R	gaggtgcagcacttcaatatagactgacccTTTATCATCGTCATCTTTATAATCAACCAATGC
11	primer_11_F	gctttctaagggccacagcGGTGGAGGATCCGGGAGC
	primer_11_R	gtggtgccttggcccaatgcTTTATCATCGTCATCTTTATAATCAACCAATGC
12	primer_12_F	gctgcttccggagccagagGGTGGAGGATCCGGGAGC
	primer_12_R	agcacgacgcctttcgtagcTTTATCATCGTCATCTTTATAATCAACCAATGC
13	primer_13_F	gactgaaggtccttttaagggcacgacaatgttggaatatGGTGGAGGATCCGGGAGC
	primer_13_R	tctttaatggcatcgaccgtaagttggagagccgtcgctgcTTTATCATCGTCATCTTTATAATCAACCAATGC
14	primer_14_F	caactcctcatgcagtctacaGGTGGAGGATCCGGGAGC
	primer_14_R	aagttctgtgacagcattgttTTTATCATCGTCATCTTTATAATCAACCAATGC
15	primer_15_F	gacaaccatccctgcaGGTGGAGGATCCGGGAGC
	primer_15_R	gattgaggcgtagtcccTTTATCATCGTCATCTTTATAATCAACCAATGC
16	primer_16_F	ggaaatcactctcttgtgcggcgaaatcttatatGGTGGAGGATCCGGGAGC
	primer_16_R	tgcggcaggctagatggcgtaactgccgtagtagtTTTATCATCGTCATCTTTATAATCAACCAATGC
17	primer_17_F	acggcactgccgaagtccatctacGGTGGAGGATCCGGGAGC
	primer_17_R	atgcttgtcagtggtctgttgtggTTTATCATCGTCATCTTTATAATCAACCAATGC
18	primer_18_F	caacaaatacgctaaggacaatccgaaagagatcgtgcttGGTGGAGGATCCGGGAGC
	primer_18_R	caggtcttgacagctccgacgaagtcgatgggcgtagccgcTTTATCATCGTCATCTTTATAATCAACCAATGC
19	primer_19_F	tccacaaggcaccacccttGGTGGAGGATCCGGGAGC
	primer_19_R	agctgcagcacgacgccggcTTTATCATCGTCATCTTTATAATCAACCAATGC
20	primer_20_F	gttacctaagaaattcGGTGGAGGATCCGGGAGC
	primer_20_R	ggaagctgcaaaataatTTTATCATCGTCATCTTTATAATCAACCAATGC
21	primer_21_F	agagatttacctggggctgacagagaactttttttGGTGGAGGATCCGGGAGC
	primer_21_R	ttgtcgtactgtaatacaattgcgccgttctccacTTTATCATCGTCATCTTTATAATCAACCAATGC
22	primer_22_F	gatttagtacagggtaccGGTGGAGGATCCGGGAGC
	primer_22_R	gcctacaatggcaccattTTTATCATCGTCATCTTTATAATCAACCAATGC
23	primer_23_F	CGCTTTCTAAtGGCCACAGCG
	primer_23_R	TGGTGCCTTGGCCCAATG
24	primer_24_F	CACCACGCTTaacaatGGCCACAGCG
	primer_24_R	CCTTGGCCCAATGCTTTAT
25	primer_25_F	tcttttgaacgacaagcattcaGGTGGAGGATCCGGGAGC
	primer_25_R	gctccctgagtcagaaagaatgtTTTATCATCGTCATCTTTATAATCAACCAATGC
26	primer_26_F	attaaataacgttcatagtGGTGGAGGATCCGGGAGC
	primer_26_R	gtagtaccctggcccaaggcTTTATCATCGTCATCTTTATAATCAACCAATGC
27	primer_27_F	tgcgaacggtacgattcatgatcgttccccattcGGTGGAGGATCCGGGAGC
	primer_27_R	tgacgacctttcagtgtcgtgccttgagataacgcTTTATCATCGTCATCTTTATAATCAACCAATGC
28	primer_28_F	tttgtcgaatgtacactccGGTGGAGGATCCGGGAGC
	primer_28_R	gtggtaccctggccaagagcTTTATCATCGTCATCTTTATAATCAACCAATGC
29	primer_29_F	ctcgaacaacaccgttcgcgaccgtactccgtacGGTGGAGGATCCGGGAGC
	primer_29_R	tgccctttagacagagttgtcccttggcccaaagcTTTATCATCGTCATCTTTATAATCAACCAATGC
**eGFP-fusion constructs**		
30	primer_30_F	gctttctaagggccacagcAGCGGAAGTGGAGATTATAAAGATGAC
	primer_30_R	gtggtgccttggcccaatgcGGAGCTCCCGGATCCTCC
31	primer_31_F	ctcgaacaataccgtccgcgatcggactccgtacAGCGGAAGTGGAGATTATAAAGATGAC
	primer_31_R	tgacccttgctcaaagtagtaccctgcccaagcgcGGAGCTCCCGGATCCTCC
