# Supplementary figures and images for: Evidence for deleterious effects of immunological history in SARS-CoV-2
Source: PLoS One. 2022 Aug 24;17(8):e0272163. doi: 10.1371/journal.pone.0272163 (PMC9401162; doi:10.1371/journal.pone.0272163)

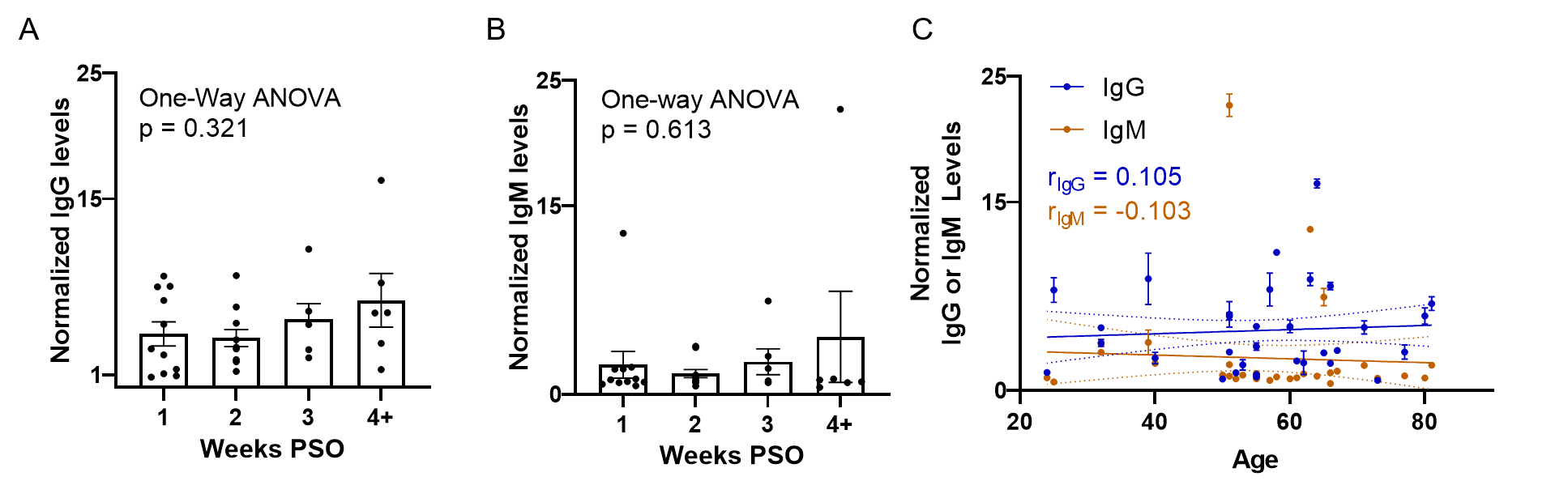

Supplement: S1 Fig — ELISA of αEp9 (A) IgG and (B) IgM levels in αEp9(+) patients (n = 34) from plasma collected at the indicated time periods post-symptom onset (PSO). Statistical analysis was conducted using one-way ANOVA, ad hoc Tukey test. Error bars represent SEM. (C) ELISA results of αEp9 IgG and IgM levels of each αEp9(+) patient displayed relative to patient age. Pearson’s correlation coefficient, r, and the 95% confidence intervals depicted as dotted lines, demonstrated no correlation between age and αEp9 Ab levels. (TIF) [file pone.0272163.s004.tif]

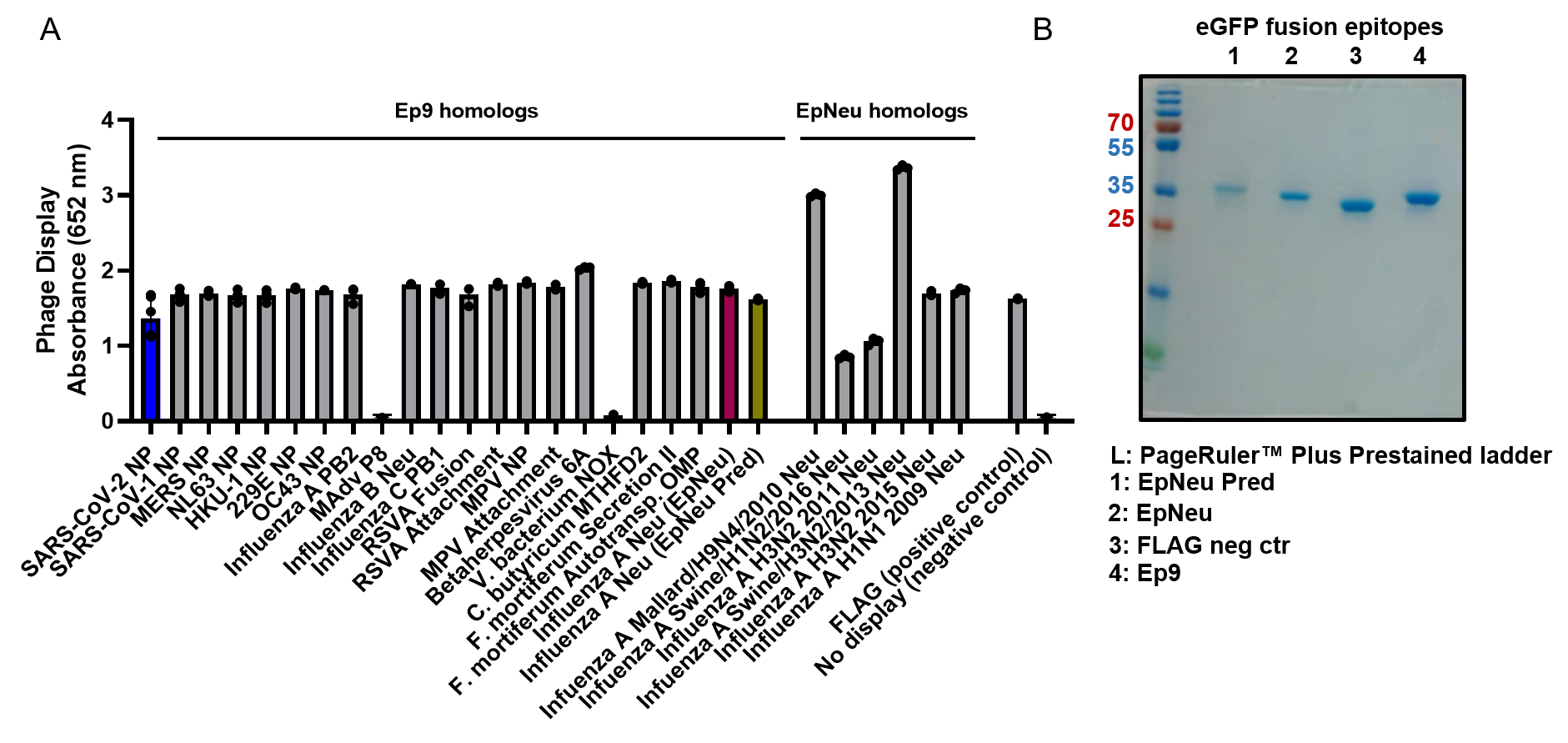

Supplement: S2 Fig — (A) ELISA demonstrating the display of N-terminal FLAG-tagged potential epitopes fused to the N-terminus of the P8 coat protein. Immobilized αFLAG Abs in microtiter wells bind the displayed FLAG-tag and epitope, and binding is detected with αM-13-HRP Abs as usual. Phage with no epitope displayed provide the negative control. Epitopes for mastadenovirus protein (mAdV) P8 and V. bacterium NADH oxidoreductase (NOX) did not display on the phage surface. Error bars represent SD values. (B) 10% SDS-PAGE gel stained with Coomassie Blue shows His-tag affinity-purified and buffer-exchanged eGFP-fused epitopes, EpPred, EpNeu, FLAG negative control and Ep9. (TIF) [file pone.0272163.s005.tif]

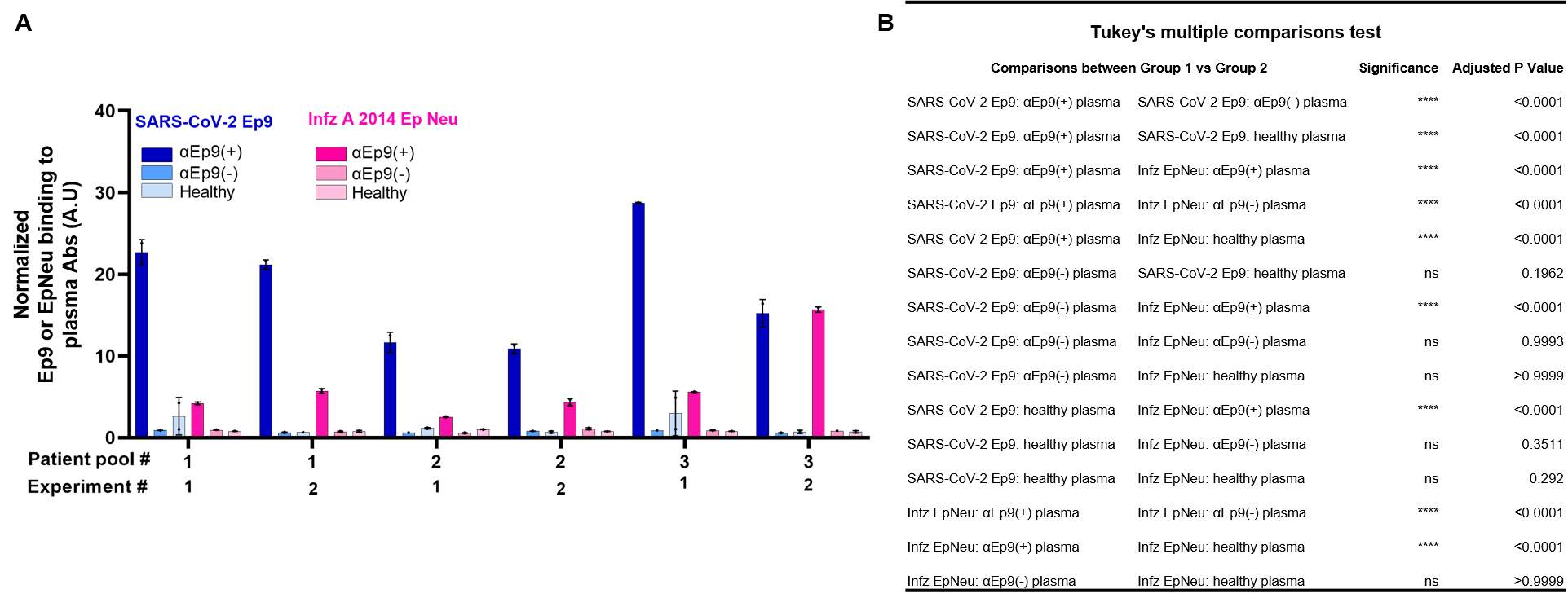

Supplement: S3 Fig — (A) Data showing the repeatability of ELISAs examining binding of phage-displayed EpNeu within a single set of pooled plasma from five αEp9(+) patients, or five αEp9(−) patients. The pooled plasma from healthy individuals was an additional negative control. Each experiment was conducted in duplicate as shown by the error bars (SD). Dots represent the actual signal from each individual ELISA well. Experiment #1 represents the same data shown in Fig 1D, with further details of duplicates. Experiment #2, represents the same pools of patients as experiment #1 but in a separate independent experimental replicate. (B) Two-way ANOVA ad hoc Tukey test was conducted where all the technical replicates from the two experiments were grouped together and compared. Significant differences are denoted by an asterisk (*) and the corresponding p-values are shown. Plasma Abs binding to EpNeu is significantly higher in the Ep9(+) patient pool compared to the Ep9(-) and healthy patient pools. (TIF) [file pone.0272163.s006.tif]

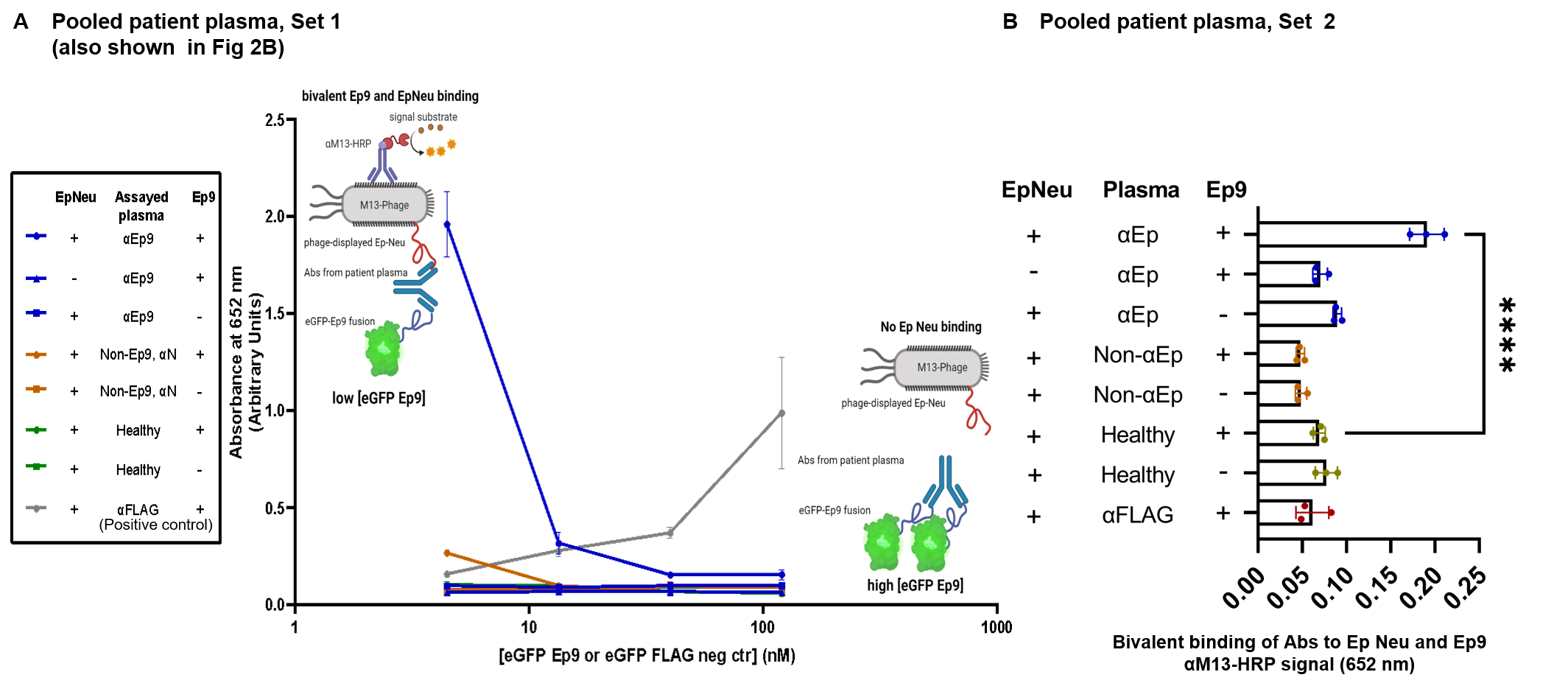

Supplement: S4 Fig — (A) Sandwich ELISA testing the binding of Abs from the pooled plasma of five αEp9(+) patients, five αEp9(-) patients with other αNP Abs and healthy individuals. This experiment examines bivalent binding to various doses of immobilized eGFP-fused Ep9 epitope (concentrations of 120, 40, 13 or 4 nM) and phage-displayed EpNeu in solution. The data shows that Abs from αEp9(+) patients, but not αEp9(−) or healthy individuals, bivalently bind both EpNeu and Ep9. The positive control (αFLAG 1:2000 fold dilution) at 100 nM eGFP demonstrates concentrations appropriate for bivalent binding to immobilized and in-solution tags. The schematic diagram illustrates the binding observed for bivalence in αEp9 Abs, where the antibody bridges plate-bound eGFP at its high concentrations. Therefore, Fig 2 in the main text uses 4 nM of eGFP Ep9 coated on the plate, and the FLAG positive control uses eGFP at 100 nM. Error bars represent SD. (B) A second set pooled plasma from five different αEp9(+) or αEp9(−) patients were tested for bivalent Ab binding. This pool was only surveyed at one dose, in which 2 nM eGFP Ep9 was coated on the ELISA plate. Bivalent binding of αEp9 Abs both Ep9 and EpNeu was exclusively observed in this αEp9(+) patient pool over background levels. As shown in panel (A) dose-dependence, the αFLAG positive control binds poorly at this concentration of coated Ep9. (TIF) [file pone.0272163.s007.tif]

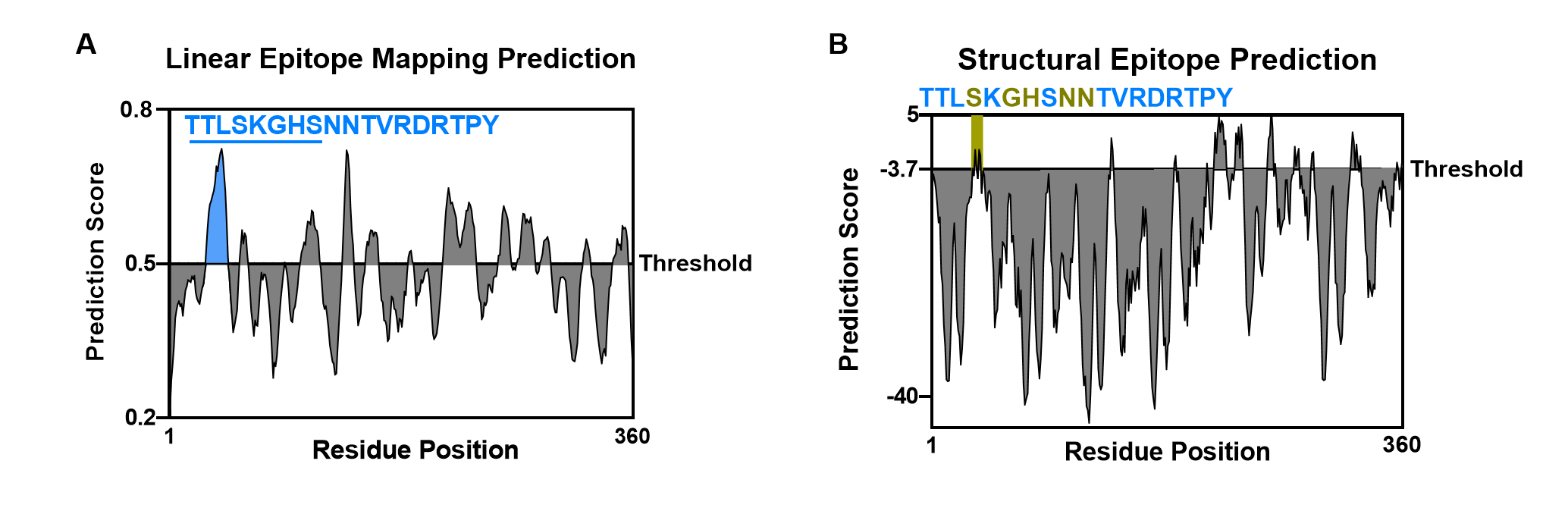

Supplement: S5 Fig — (A) Linear epitope mapping prediction of the Influenza A 2014 H3N2 using Bepipred 2.0[16] demonstrates high prediction scores in a region spanning 18 residues, which includes eight residues from EpNeu (underlined). The additional ten predicted residues were included as part of an extended epitope termed EpPred. (B) Structural epitope mapping, using Discotope 2.0[17], of the modelled neuraminidase protein from Influenza A 2014 H3N2 (SWISS-model[14],(3 predicts an epitope of five residues. These were captured by EpPred, including three found in EpNeu. (TIF) [file pone.0272163.s008.tif]

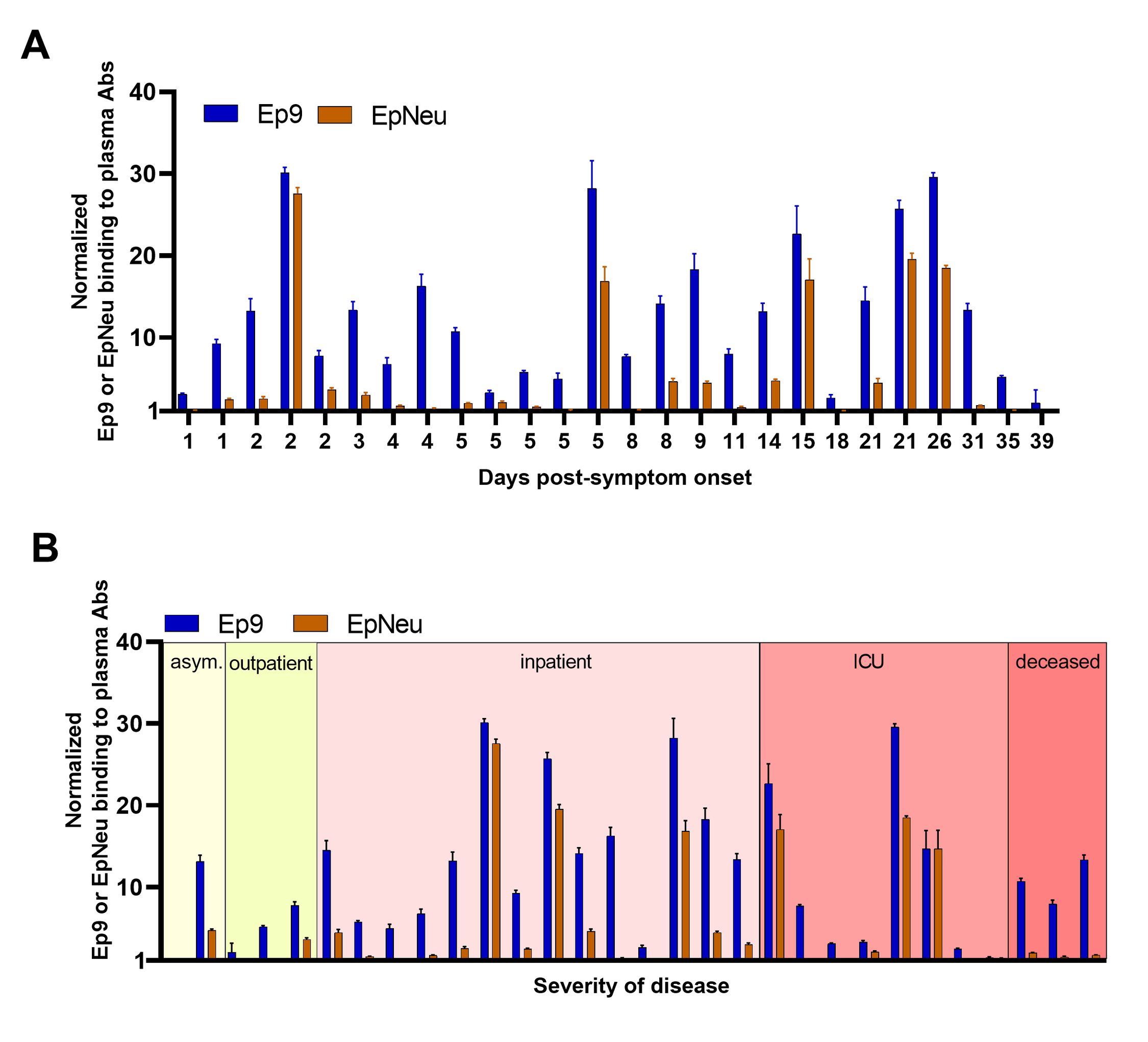

Supplement: S6 Fig — (A) Phage ELISA using 29 previously tested αEp9(+) COVID-19 patients and each sample’s days PSO. The ELISA depicts binding of patient plasma Abs to SARS-CoV-2 epitope, Ep9 (blue), or the influenza A neuraminidase epitope, EpNeu (orange). The data is normalized by fold over binding by phage with no displayed epitopes. (B) Normalized levels of phage-displayed Ep9 and EpNeu binding to plasma-coated wells from individual αEp9(+) patients (n = 26) relative to disease severity (asymptomatic, outpatient, inpatient, ICU, and deceased). (TIF) [file pone.0272163.s009.tif]
