# Supplementary material for: Evidence for deleterious effects of immunological history in SARS-CoV-2
Source: PLoS One. 2022 Aug 24;17(8):e0272163. doi: 10.1371/journal.pone.0272163 (PMC9401162; doi:10.1371/journal.pone.0272163)
Supplement: Attachment — Submitted filename: PLoSOne_Reviewer Response-040622.pdf [file pone.0272163.s010.pdf]

Proposal link

We thank the Editor and Reviewers for their time and insights. With this revision, we have responded to all of their suggestions and comments. The resulting manuscript benefits from the changes and is significantly stronger.

Journal Requirements:

When submitting your revision, we need you to address these additional requirements.

1. Please ensure that your manuscript meets PLOS ONE's style requirements, including those for file naming. The PLOS ONE style templates can be found at

https://journals.plos.org/plosone/s/file?id=wjVg/PLOSOne_formatting_sample_main_body.pdf and

https://journals.plos.org/plosone/s/file?id=ba62/PLOSOne_formatting_sample_title_authors_affiliations.pdf

This revision includes changes to the manuscript's format as required by the guidelines.

2. Thank you for stating the following in the Acknowledgments/ Funding Section of your manuscript:

We gratefully acknowledge the support of the UCI COVID-19 Basic, Translational and Clinical Research Fund (CRAFT), the Allergan Foundation, and UCOP Emergency COVID-19 Research Seed Funding. A.M.S. thank the Minority Access to Research Careers (MARC) Program, funded by the NIH (GM-69337). J.L.R.-O. was supported by the National Center for Research Resources and the National Center for Advancing Translational Sciences from the NIH (TR001414).

Please note that funding information should not appear in the Acknowledgments section or other areas of your manuscript. We will only publish funding information present in the Funding Statement section of the online submission form.

Please remove any funding-related text from the manuscript and let us know how you would like to update your Funding Statement. Currently, your Funding Statement reads as follows:

G.A.W - UCI COVID-19 Basic, Translational and Clinical Research Fund (CRAFT), the Allergan Foundation, and UCOP Emergency COVID-19 Research Seed Funding. A.M.S. - Minority Access to Research Careers (MARC) Program, funded by the NIH (GM-69337,

https://www.nigms.nih.gov/training/MARC/Pages/USTARAwards.aspx). J.L.R.-O. was supported by the National Center for Research Resources and the National Center for Advancing Translational Sciences from the NIH (TR001414, https://ncats.nih.gov/funding/). The funders had no role in study design, data collection and analysis, decision to publish, or preparation of the manuscript.

We have removed funding information and confirm that the statement highlighted above is accurate. No further changes will be made.

Please include your amended statements within your cover letter; we will change the online submission form on your behalf.

Done.

3. Please note that in order to use the direct billing option the corresponding author must be affiliated with the chosen institute. Please either amend your manuscript to change the affiliation or corresponding author, or email us at plosone@plos.org with a request to remove this option.

The corresponding author (Gregory Weiss) is a full-time researcher at UC Irvine, at the affiliated institution.

Additional Editor Comments:

Several reviewers raised similar points regarding use of pooled sera and relatively small sample size. The requested revisions were alternatively listed as "major" or "minor" depending on the reviewer. Please do your best to address each reviewer's concerns, especially for the common points of concern.

Thank you very much for giving us an opportunity to respond, which we have done comprehensively as described below.

[Note: HTML markup is below. Please do not edit.]

Reviewers' comments:

Reviewer's Responses to Questions

Comments to the Author

1. Is the manuscript technically sound, and do the data support the conclusions?

The manuscript must describe a technically sound piece of scientific research with data that supports the conclusions. Experiments must have been conducted rigorously, with appropriate controls, replication, and sample sizes. The conclusions must be drawn appropriately based on the data presented.

Reviewer #1: Yes

Reviewer #2: No

Reviewer #3: Yes

---

2. Has the statistical analysis been performed appropriately and rigorously?

Reviewer #1: Yes

Reviewer #2: Yes

Reviewer #3: I Don't Know

---

3. Have the authors made all data underlying the findings in their manuscript fully available?

The PLOS Data policy requires authors to make all data underlying the findings described in their manuscript fully available without restriction, with rare exception (please refer to the Data Availability Statement in the manuscript PDF file). The data should be provided as part of the manuscript or its supporting information, or deposited to a public repository. For example, in addition to summary statistics, the data points behind means, medians and variance measures should be available. If there are restrictions on publicly sharing data—e.g. participant privacy or use of data from a third party—those must be specified.

Reviewer #1: Yes

Reviewer #2: Yes

Reviewer #3: Yes

---

4. Is the manuscript presented in an intelligible fashion and written in standard English?

PLOS ONE does not copyedit accepted manuscripts, so the language in submitted articles must be clear, correct, and unambiguous. Any typographical or grammatical errors should be corrected at revision, so please note any specific errors here.

Reviewer #1: Yes

Reviewer #2: Yes

Reviewer #3: Yes

---

5. Review Comments to the Author

Please use the space provided to explain your answers to the questions above. You may also include additional comments for the author, including concerns about dual publication, research ethics, or publication ethics. (Please upload your review as an attachment if it exceeds 20,000 characters)

Reviewer #1: In the current manuscript, Sen et al. provide evidence that antibodies targetting a region of SARS-CoV-2 nucleocapsid protein, previously found to be correlated with COVID-19 severity, can recognize an epitope from the neuraminidase protein of influenza A virus. In light of their results, the authors propose that existing memory from influenza infections, in particular the H3N2 strain that affected he United states in 2014, could trigger a deletereous Ab response that exacerbates COVID-19 severity.

Overall, the authors' hypotheses and aim is well presented and the results support them. He language is, for the most par, corret, as well as he structure of the manuscript. I have some minro comments

Fig. 1C: Phage binding: Why is plasma directly coated unto plates? Adsorption unto plasic is a highly unspecific process, where all proteins are going to compete for binding to plastic, making any comparison complicated. A beter approach would be to coat the antigen, apply the plasma, and then detect IgG or IgM bound (as the authors do for the antiEp9 IgG and IgM ELISAs).

The reverse phage ELISA method used in this manuscript is standard for the field. See, for example, Chen et al., Science Advances volume 6, number 14, 2020 and Lim et al.,Scientific Reports volume 9, number 6088, 2019. Preliminary experiments testing both reverse and direct ELISA methods demonstrated significantly lower background signals with the reverse phage ELISA approach when used with the commercial blocking agent, Chonblock, and therefore, our investigations were based on this standard method of analysis.

Also, why is the OD measured at different times?

L359-361 states that "Absorbance of TMB substrate was measured twice at 652 nm by UV-Vis plate reader (BioTek Winooski, VT) after 5 and 15 min of incubation." To clarify this, we have added the following to the manuscript, **L361-365** "The measurement at 5 min ensured that ELISAs with strong signals were quantified before oversaturation, and the second measurement at 15 min was collected to enhance any wells with lower level signals; the approach ensures that no comparable signals were observed in the negative controls. In ELISAs without oversaturated signals, the measurements at 15 min were used for data analysis."

Fig. 1E vs 1F: Fig. F is inroduced earlier into the text, so the panels should be switched. At the same time, the table contains an extra putative epitope not tested in panel E.

The figure order has been changed. The extra, untested putative epitope from the table has been removed.

Fig. 1, 2: Is there a reason to pool patient samples? Is it due to big differences in their Ab titers? Otherwise, plotting individual samples, even stratified by Ep9 levels, would be more informative.

Once a cross-reactive epitope was identified, individual patients were analyzed for the prevalence of the cross-reactive Ab, and a new figure Fig. 2A has been added to the paper with this data. As suggested by the reviewer, we further expanded the data in figure 2A to demonstrate Ab binding levels to both Ep9 and EpNeu for each individual patient in Fig 1G.

This revision also retains the data from pooled patient samples for the following reason (text added to the manuscript). **L139-140** "Since patient samples were collected at different time points during the patients' infection, Ab levels varied

significantly between patients. Thus, patients' samples were pooled for some assays to minimize outlier concentrations and best capture the average Ab population in patients."

Additionally, while we observed anti-Ep9 Ab levels in individual patients, it was unknown whether anti-Ep9 Abs derive from a single type of Ab or a population of Abs targeting the same large epitope. Therefore, pooling Ep9 positive patients (as in Fig. 1 C, D and E) provided an efficient approach for screening potentially slightly variant Abs to determine if any patients within the pool had cross-reactive Abs.

Fig. 2: A good addition would be to run a competition study to demonstrate that Ep-Neu and Ep9 share the same paratope. Also, the axis naming is no very clear (2A), or descriptive (2B), or consistent (AE, e.g. Bound serum IgG (OD 652 nm) and Epitope concentration (µM)). The minute differences between EpPred and EpNeu should be evaluated with a technique to evaluae (e.g. SPR) to conclude anything (L205–207).

Fig 2 A and 2E cannot have similar axes as they are two different types of ELISAs. Fig 2A (now 2B) is a phage ELISA, using a specific concentration of epitope-displayed phage and the readout is anti-M13-HRP, normalized to no display controls. Here the values have to be normalized to negative controls for facile comparisons between individual patients. These negative control values and individual patient data have now been included as the new Fig 2A. Fig 2E (now 2F), on the other hand, demonstrates dose-dependent binding of these epitopes for comparison of their EC50s. Therefore, instead of using multivalent phage-displayed epitopes, eGFP-fused epitopes are used. For the latter, the readout is anti-IgG-HRP signal, and the negative control has been shown.

The positive signal in the bivalent Ab binding in the ELISA on Fig 2B demonstrates that a single antibody binds both epitopes Ep9 and Ep Neu, thereby confirming that they do in fact share the same paratrope.

The suggestion to perform the SPR experiment is an interesting one, but unfortunately the quantities of patient samples remaining are too small for this experiment. Since we are analyzing the epitopes removed from the full-length NA protein (due to the known difficulties in overexpression, as explained in the manuscript, **L234**), the relative binding of the Abs to the different epitopes can be more relevant than the Kd values. Thus, the EC50s from the ELISAs should be sufficient for such comparisons.

To address reviewer concerns about the minute differences in binding, we have made changes to the text to more precisely describe the trends observed. In **L232-235** we have added "The longer length EpPred appears to modestly improve upon binding of EpNeu to αEp9 Abs (Fig. 2E). Thus, while αEp9 Abs may target a larger epitope of H3N2 2014 NA beyond regions homologous to Ep9, the known balkiness of full-length NA's to overexpression makes this hypothesis difficult to test[18]"

Minor comments:

L78, 83, 85, 273: Incorrect placement of commas, such as “hCoVs, NL63 and 229”, “comprised 27% of the sampled, SARS-CoV-2-infected population”, “cytokine-related, immune hyperactivity”, or “0.05%, v/v”.

L81-82: “The presence of Abs[…] have”, correct to “has”

L 152: The authors refer to H4N6 avian influenza. Do they mean H9N4?

156: A very conversational one. I would recommend a more classical way to introduce and connect the next batch of experiments.

L268: 1/5TH?

Format: Thousands separator use is not consistent (e.g. L271, 277), incorrect use of hyphens for minus emperature (minus symbol), range (n dash without preceding or trailing spaces)

The above changes have been made. We thank the reviewer for their excellent suggestions, which have strengthened our manuscript.

Reviewer #2: This manuscript investigates whether there is homology between the Ep9 epitope of the SARS-CoV-2 N protein and other proteins. The authors previously showed that individuals infected with SARS-CoV-2 that had Ep9-specific antibodies had a worse prognosis. Here, they investigate whether this could be due to antigenic imprinting and therefore search for cross-reactive epitopes. While this is an interesting hypothesis, the data do not convincingly support it due to the concerns listed below.

• In Figure 1, the data demonstrating binding to the different epitopes is done with 3 sets of pooled serum. While the differences are statistically significant, it is difficult to determine how relevant they are when only n=3 is shown and the experiment is not repeated.

To address the reviewer’s concerns we have added new Fig. S1A, which shows the two independent replicates of a pool of aEp9(+) and aEp9(-) patients, in addition to the pooled plasma from healthy patients (Sigma). The experiment includes three technical replicates for the three different pools of patients (n = 5 patients for aEp9(+) or aEp9(-)). Error bars and data points show data from the technical replicates for each experiment. Additionally, two-way ANOVA *ad hoc* Tukey of replicates from both experiments show significant increases in EpNeu binding

signal from aEp9(+) plasma Abs, but not in aEp9(-) patients. This data has also been described in the manuscript on **L155-158**.

• It would be helpful to see binding to the eGFP-Eph fusion protein by ELISA for each individual person.

Done – in the new Fig. 2A. Plasma Abs from previously confirmed aEp(+) patients were individually tested for Ep9 and EpNeu binding.

• In Figure 2A, they analyze 34 samples independently, and show that only 6/34 of the samples bind to both Eph and EpNeu. Thus, although there is a significant correlation in the values, not all Eph+ individuals are EpNeu+.

To clarify, of 34 aEp9(+) patients, 29 were independently tested for anti-EpNeu Abs. A correction was made to L166 and we have added a statement of clarification on **L370-372** "Due low sample availability for 5 patients, the plasmas from 29 patients were then used to compare IgG binding to the Ep9 and the EpNeu epitopes." Of these 29 patient samples, 16 showed significant increase in Ab binding over background. The 6 patients that the reviewer is referring to are the patients that demonstrate the highest levels of binding to both EpNeu and Ep9.

• The experiment measuring cross-reactivity between the two antigens by sandwich ELISA shows technical replicates (n=3) of 1 pool of plasma. Therefore, it is not possible to determine if there is binding to both antigens in more than one person.

The new Fig. S4B presents additional data wherein plasma from five different aEp9(+) or aEp9(-) patients were pooled and tested for the Ab-mediated bivalent interaction. This data also shows such Ab cross-reactivity. Therefore, it can be concluded that the cross-reactive binding is reproducible amongst the patient population and not due to a single patient.

• The conclusions that cross-reactivity between the epitopes could result in antigenic imprinting are not supported by the data. At best, the data show that there may be cross-reactivity between these similar epitopes. However, the fact that one amino acid substitution in the NP protein of other influenza strains completely blocks binding, rather than a reducing binding raises the question of whether the binding to the EphNue is real. One would also expect more cross-reactivity with that epitope in HKU1 and OC43 as there are only 1-2 amino acid differences between this epitope in these viruses and SARS-CoV-2.

Evidence suggests that both broad and narrow antibodies are generated against the influenza virus. In the case of narrow Abs, single site mutations are sufficient for viruses to escape neutralization. This is further investigated in Doud, MB et al. (How single mutations affect viral escape from broad and narrow antibodies to H1 influenza hemagglutinin, Nature Communications, volume 9, article number 1386, 2018).

Despite there being high homology in EpNeu region between SARS-CoV-2 and HKU1 or OC43, it does not contain the P**K**G motif but instead has the sequence P**Q**G. As such we observe that Ep9 patients do not have Abs that target this region. This provides further evidence that the single amino acid is important for Ep9 Ab recognition.

• Finally, if cross-reactivity to EphNeu was causing antigenic imprinting and negatively impacting generation of antibodies specific for SARS-CoV-2 Eph9 in some individuals, one would expect that you would detect EpNeu reactivity in some healthy controls. In other words, if prior exposure to the 2014 influenza was responsible for the variability in SARS-CoV-2 infection, then you would expect to see reactivity in the general population prior to SARS-CoV-2 infection.

As stated on **L345**, healthy samples were commercially purchased from pooled healthy patients Sigma-Aldrich. As such their medical records or dates of collection were not available. Therefore, it's hard to know whether these healthy patients would likely have been exposed to the 2014 EpNeu infection. Additionally, the Ep9 Abs in COVID-19 patients are prevalent in detectable amounts in their serum due to an active response against infection; healthy patients who may have been previously exposed to the H3N2 2014 influenza strain and generated anti-EpNeu Abs could still have memory B cells, but may not have detectable levels of Abs in their serum.

Reviewer #3: Sen et. al., report an interesting study examining the hypothesis that some severe Covid-19 cases may be the result of antigenic interference. They propose a mechanism where a pre-existing antibody response to a H3N2 influenza infection, resulted in cross-reactivity to the SARS-COV2 Ep9 epitope. This is an interesting and compelling hypothesis. The authors carry out ELISA based experiments to demonstrate that Ep9 antibody containing plasma cross-reacts with a homologous epitope found in the H3N2 neuraminidase. Having previously established a correlation between Ep9 containing sera and Covid-19 severity, a picture emerges whereby previous infection by H3N2 may explain increased Covid-19 severity in Ep9+ patients.

In general, the study is well executed and appropriate controls are included. The manuscript is well written, and this reviewer views this research favorably.

We thank the reviewer for their support.

The main issue with the study is related to the relatively small sample size. For all the ELISA studies, pooled plasma (n= 3) from 5 individuals was used. The effect size is rather pronounced, as the ep9- and negative controls have essentially zero binding. The reviewer lacks the expertise to make the judgment regarding if this sample size is sufficient to draw a definitive conclusion. To this end, either the authors need to increase their sample size, or provide sound reasoning why such a

small sample size is sufficient or carefully qualify their results in light of the small sample size.

The individual data of all patients have now been added to the manuscript as new Fig 2A. These ELISAs demonstrate that patients that have Abs against Ep9 also bind the EpNeu epitope. The pooled sample data are initially used to screen for cross-reactivity against multiple possible epitopes and secondly to support conclusions that are observed from epitope binding observed in individual patients.

Some minor points that could use additional clarification:

1. The authors describe an initial plasma collection from 34 individuals, but then make use of pooled plasma for their experiments. Each pool contains plasma from 5 donors. The authors should explain the rationale for using pooled plasma as opposed to plasma from individuals.

The experiments were designed to work with very limited patient samples. The pooled sample data are initially used to screen for cross-reactivity against multiple possible epitopes and secondly to support conclusions that are observed from epitope binding observed in individual patients. See Fig 2A demonstrating Ep9 and EpNeu binding of individual patients. Explanation added to manuscript **L142-144.** Experiments such as the bivalent ELISA were not possible for individual patients as the epitope concentrations coated to the plate had to be optimized for the levels of Abs in each individual patient to allow for bivalent binding to each type of epitope. For pooled patients,it was speculated that the average amount of Abs in each pool would be similar and that the repeated optimization would not be required. An explanation was added to manuscript in **L180-184.**

2. It is somewhat unclear what the number of healthy donors was for their negative control. Is it also 3 samples of pooled plasma, each with 5 donors?

As stated on **L345**, healthy samples were commercially purchased pooled from healthy patients Sigma-Aldrich.

3. Page 2 lines 57+58. - The statement is also somewhat unclear. Do they mean they discovered a particular antibody or do they mean a population of antibodies in sera (i.e. antibodies?)

“Since the study focuses on identifying epitope binding traits of Abs that are upregulated in SARS-CoV-2 positive patients,we cannot discern between a single Ab or a population of Abs of a certain serotype with the same binding profile. Additionally, we observe that the Ep9 epitope is targeted by IgG and IgM antibodies, suggesting that multiple antibodies with similar binding profiles may exist in SARS-CoV-2 patients. Therefore, we refer to the anti-Ep9 epitopes as a population of Abs in sera” (**L100-106**).

Again, we are grateful to the reviewers for their insights and help strengthening the manuscript.
