# Supplementary material for: Evidence for deleterious effects of immunological history in SARS-CoV-2
Source: PLoS One. 2022 Aug 24;17(8):e0272163. doi: 10.1371/journal.pone.0272163 (PMC9401162; doi:10.1371/journal.pone.0272163)
Supplement: Attachment — Submitted filename: PLoSOne Responses-to-Reviewers-062222.pdf [file pone.0272163.s011.pdf]

6. Review Comments to the Author

Reviewer #1: (No Response)

Reviewer #2: The authors have addressed many of the concerns raised in the previous review. However, I am still not convinced that antibodies generated by a previous influenza infection are having a negative impact on the immune response to SARS-CoV-2 as there is no evidence that antibodies in these samples bind to an epitope that elicits an antibody response after influenza exposure. Additionally, there is no evidence that antibodies in these samples bind to intact NA protein or epitopes that may be presented by infected cells. Moreover, antibodies that impact the immune response to a pathogen via imprinting, should be detected early after exposure and that information is not presented.Since there may not be sufficient number or volume of samples, the authors could modify their conclusions to indicate that their data are consistent with their overall hypothesis, rather than these data support their hypothesis.

The reviewer makes excellent points. We agree and have fully addressed their points with this revision, as discussed below (e.g., we have modified our conclusions to state "consistent with the hypothesis," not "supporting the hypothesis").

Showing the antibody reactivity in each individual in Fig 2A is very helpful. Since the authors make the point that plasma was collected at different times, which may contribute to the variability in antibody levels, it would be very informative to include the day that the sample was collected in Fig 2A. Since the data are presented in a bar graph, the samples could be arranged by day after symptom onset, rather than patient number. This would enable you to assess whether individuals that had increased levels of Ep9 antibodies early also had antibodies reactive to EpNeu, which would support the imprinting hypothesis. If the EpNeu antibodies are generated by a previous infection and they have an impact on SAR-CoV-2 infection, you would expect to see binding to EpNeu early

Records for days PSO were available for 26 patients. So, we have analyzed the relationship between days PSO and αEp9 Ab cross-reactivity as suggested by the reviewer in a new **Figure S6A**. The results are described within the manuscript in Lines 458-463, as follows: "Next, we analyzed Ep9 and EpNeu binding by αEp9 IgGs relative to days PSO (S6A Fig). Cross reactive αEp9 IgGs were observed within one day PSO. The observation is consistent with the imprinting hypothesis, whereby mature IgGs from a previous infection would be present early in the course of the infection. Though low levels of early αEp9 IgGs bound without EpNeu cross reactivity were observed in one patient at one day PSO, this observation could result from EpNeu binding below the level of detection; αEp9 Ab binds at lower affinities to EpNeu, for example."

We thank the reviewer for their excellent suggestion to look at this issue, as it adds insight to the paper.

Line 237 – the results do not necessarily support the hypothesis as it is still not clear whether this epitope is presented during infection or even in the full-length protein. If the full-length NA can't be made in bacteria, it can be expressed in other cell types.

These limitations to the study have been described in the discussion section. While we were unable to test antibody binding to the full-length neuraminidase protein, both sequence and structural predictions predict that the EpNeu site is likely an epitope region (S5 Fig). Additionally, the EpNeu site is adjacent to a known escape mutation, suggesting that the EpNeu site is critical to antibody response. We 100% agree with the reviewer's comments that these predictions are ***consistent*** with the overall hypothesis and have modified our statements accordingly.

The new line 237-238 was revised to state the following,"Taken together, the results are consistent with the hypothesis that αEp9 Abs found in severe COVID-19 can result from AIN with H3N2 influenza A virus."

Thank you for clarifying that 16/29 patients with antibodies reactive against Ep9 also had antibodies reactive against EpNeu. While this is greater than the 6 individuals that have high EpNeu levels, the fact remains that not all Ep9 antibodies cross-react to EpNeu. This should be considered in the discussion. Is there a stronger correlation with disease severity with EpNeu binding compared to Ep9?

We thank the reviewer for the suggestion to examine a correlation with severity. We have conducted an analysis of disease severity relative to EpNeu cross-reactivity, which is represented as S6B Fig. While we do not find a direct correlation between the disease severity and crossreactivity, a subset of patients with severe disease outcomes (hospitalized or admitted to the ICU) with high levels of plasma Ab binding do show greater levels of cross-reactivity (>50%) suggesting impaired affinity maturation.

Lines 464-493 have been added to the discussion as follows, "Analysis of αEp9 IgG cross reactivity and disease severity demonstrated that cross reactive antibodies were observed in patients presenting with all levels of severity (asymptomatic, outpatient, inpatient, ICU admittance, or deceased) (**S6B Fig**). While EpNeu binding in most patients was drastically lower than binding to Ep9, a subset of hospitalized or ICU admitted patients demonstrated αEp9 Abs binding to EpNeu and Ep9 at comparable levels (>50%). Such similar Ab binding levels to both Ep9 and EpNeu are not observed in patients with less severe outcomes (i.e., patients who were asymptomatic or experienced only outpatient visits). However, 86% of the samples tested in this study were from hospitalized and admitted to the ICU patients. Similar levels of Ab binding to both Ep9 and EpNeu in the subset of hospitalized and ICU-admitted patients could suggest impaired affinity maturation in patients with more severe outcomes. Impaired Ab affinity maturation have been previously shown to correlate with COVID-19 severity(23,24). While multiple factors may lead to disease severity during COVID-19, our results suggest that a reliance on high levels of imprinted influenza Abs by a subset of COVID-19 patients could be indicative of a less effective immune response and consequently more severe disease outcomes."

Minor points:

• Fig 1C – Is that IgG, IgM or total Ig?

Changes made to Line 124-126: ELISAs examined binding of phage-displayed potential OAS epitopes to total Ig from three sets of pooled plasma from five αEp9(+) patients, or five αEp9(−) patients.

• I think that lines 231-234 refer to Fig 2F, not Fig 2E as indicated.

Good catch! We have changed this to Fig 2F.

In summary, we have modified the manuscript to respond to all of the Reviewer's excellent suggestions. The resultant manuscript has been strengthened by the reviewer's insights and we thank them for their supportive help.

Reviewer #3: I am satisfied with the author's responses to all concerns raised by the reviewers. I support acceptance of the manuscript as is.

---

7. PLOS authors have the option to publish the peer review history of their article (what does this mean?). If published, this will include your full peer review and any attached files.

If you choose "no", your identity will remain anonymous but your review may still be made public.

Do you want your identity to be public for this peer review? For information about this choice, including consent withdrawal, please see our Privacy Policy.

Reviewer #1: **Yes:** Rafael Bayarri-Olmos

Reviewer #2: No

Reviewer #3: No
